# Can the antral follicular count modulate the gene expression of bovine oviducts in Aberdeen Angus and Nelore heifers?

**DOI:** 10.1371/journal.pone.0202017

**Published:** 2018-08-29

**Authors:** Patricia Kubo Fontes, Ronaldo Luis Ereno, André Rebello Peixoto, Robson Francisco Carvalho, Wellerson Rodrigo Scarano, Luzia Aparecida Trinca, Ciro Moraes Barros, Anthony César de Souza Castilho

**Affiliations:** 1 Departament of Pharmacology, Institute of Biosciences, Universidade Estadual Paulista (UNESP), Botucatu, São Paulo, Brazil; 2 Departament of Morphology, Institute of Biosciences, Universidade Estadual Paulista (UNESP), Botucatu, São Paulo, Brazil; 3 Departament of Biostatistic, Institute of Biosciences, Universidade Estadual Paulista (UNESP), Botucatu, São Paulo, Brazil; 4 Departament of Animal Science, University of Western São Paulo (UNOESTE), Presidente Prudente, São Paulo, Brazil; University of Florida, UNITED STATES

## Abstract

The number of visible ovarian antral follicles (antral follicle count—AFC) is repeatable in bovine individuals, but highly variable between animals, and with differences between *Bos taurus* and *Bos indicus* breeds. Several studies have tried to determine the correlation between AFC and increased fertility in cattle. While the impacts of AFC on embryo production, hormonal levels, and pregnancy rates have been described, the molecular effects of AFC on bovine oviducts have not yet been investigated. Here, the aim was to investigate the impact of breeds, such as Aberdeen Angus and Nelore heifer with high or low AFC, on abundance of transcripts and protein related to oviductal transport, sperm reservoir formation, monospermy control, and gamete interaction in the oviducts. In summary, the ovulation side was the major factor that affected transcript abundance on bovine oviducts. However, a discreet effect among AFC and cattle breeds was also observed. Based on this, we concluded and reinforced here that differential microenvironments between ipsilateral and contralateral oviducts have a major effect on modulating the transcripts related to oviductal transport, sperm reservoir formation, monospermy control, and gamete interaction. However, we cannot exclude that there is minimal effect of AFC or breed on regulation of some genes (such as *AGTR1*, *ACE1*, *FUCA1*, and *VEGFA*) in bovine oviducts.

## Introduction

Several studies have investigated the relationship between the ovarian antral follicle count (AFC) and bovine fertility [1, 2]. There is evidence that AFC is highly variable among different animals, but it is constant within the same animal during their reproductive life [3–6]. This allows for distinguishing between animals with a high (HFC) and low follicle count (LFC). A high number of follicles per wave is directly associated with an increased efficiency in reproductive biotechnology techniques, such as embryo transfer, *in vitro* embryo production, and ovarian superstimulation [5, 7]. On the other hand, low AFC is associated with impaired fertility, with reduced conception rates, longer calving to conception intervals [2], and lower competence of oocyte nuclear maturation [1].

The reproductive differences between *Bos taurus indicus* and *Bos taurus taurus* cattle are mostly known. *Bos taurus indicus* cows recruit more follicles per follicular wave than *Bos taurus taurus* [8–10] and the number of follicles per wave for animals with HFC or LFC differs between each genetic group; when comparing HFC animals of both breeds, there are greater numbers of follicles in *Bos taurus indicus* than in *Bos taurus taurus*. Similarly, LFC animals in *Bos taurus taurus* populations present lower numbers of follicles than *Bos taurus indicus* [10]. Greater total uterine luminal protein levels were also demonstrated in *Bos taurus taurus* (Angus) when compared to *Bos taurus indicus* (Brahman) cows [8], while the protein content was less in Angus heifers with LFC than for heifers in the HFC group, suggesting the uterine environment for Angus with HFC is more conducive to supporting early embryonic survival [11].

Oviductal functions are related to successful embryo production and conception [12, 13]. The oviduct is responsible for providing an ideal microenvironment for final gamete maturation and transport, fertilization, and early embryo development through the infundibulum, ampulla, and isthmus segments [14]. The infundibulum picks up the cumulus-oocyte complexes (COC) and transports them to the ampulla [15], where fertilization and early embryo development occurs [16]. The isthmus plays a key role in the formation of a sperm reservoir, capacitation, and hyperactivation [17–19].

To guarantee the success of reproductive function, the oviduct has a temporal and spatial organization per segment [20, 21]. In the follicular phase, the epithelium of the infundibulum and ampulla exhibits numerous and prominent ciliated cells [22]. The oviductal ciliary beat frequency (CBF) is directed toward the uterus [23] and stimulated by prostaglandin E_2_ [24], angiotensin II [25], and estradiol [26], and inhibited by progesterone [26, 27]. Isthmus ciliary cells are also involved in sperm reservoir formation, and the ciliated cells express annexins (sperm receptors) on their surfaces [28], which are released by α-L-fucosidase (FUCA) when it is time to transport sperm to the fertilization site [29].

Associated with prostaglandins and angiotensin systems, vascular endothelial growth factors and endothelin are involved in the transport activity of smooth muscle contraction. The association of CBF and smooth muscle contraction gives the oviduct a bidirectional transport ability [30]. The oviductal epithelium also consists of secretory cells, responsible for oviductal fluid production [31]. The secretory cells are present starting from the pre-fertilization period, or they are induced by the embryo to ensure an optimal microenvironment for monospermy control, gamete interaction, and nutrition for the first days of embryo development [16]. Thus, the oviductal role for animal fertility is evident.

Since fertility has been associated to AFC, and AFC classification differs between cattle breeds. Here, we tested our hypothesis that cattle breeds and AFC could influence the genes and protein levels in the bovine oviduct. For this, we compare transcripts and protein levels related to oviductal transport, sperm reservoir formation, monospermy control, and gamete interaction in the infundibulum, ampulla, and isthmus samples collected 24 hours after ovulation time in both ipsilateral and contralateral bovine oviducts from Nelore and Aberdeen Angus heifers with HFC and LFC.

## Materials and methods

All animal procedures were approved by the Ethics and Animal Handling Committee of the Universidade Estadual Paulista (UNESP), Botucatu, São Paulo, Brazil, certificate #378.

### Animal selection

This study was conducted on a farm located in Ribeirão do Sul (São Paulo, Brazil; latitude −22° 47′ 03′′; longitude −49° 56′ 01′′; altitude 479 m). Heifer selection and AFC group classification were previously described by Loureiro *et al*. [32]. Briefly, using ultrasound examination (US, Mindray, 5–10 MHz, China), the total number of follicles was determined in 100 Aberdeen Angus and 100 Nelore heifers in a random day of estrus cycle. All selected heifers were cycling (with CL presence) and had no follicles greater than 5 mm. Then, these heifers were synchronized with two doses of PGF2α 11 days apart. Four days after the second PGF2α (approximately 24 hours after follicle recruitment), another US evaluation was performed to confirm the total number of follicles in each heifer. Considering the mean of AFC ± standard deviation (SD) in each breed, the heifers were classified into two groups: LFC (animals with a total number of follicles below the mean—SD) and HFC (animals with a total number of follicles above the mean + SD).

**Fig 1:**
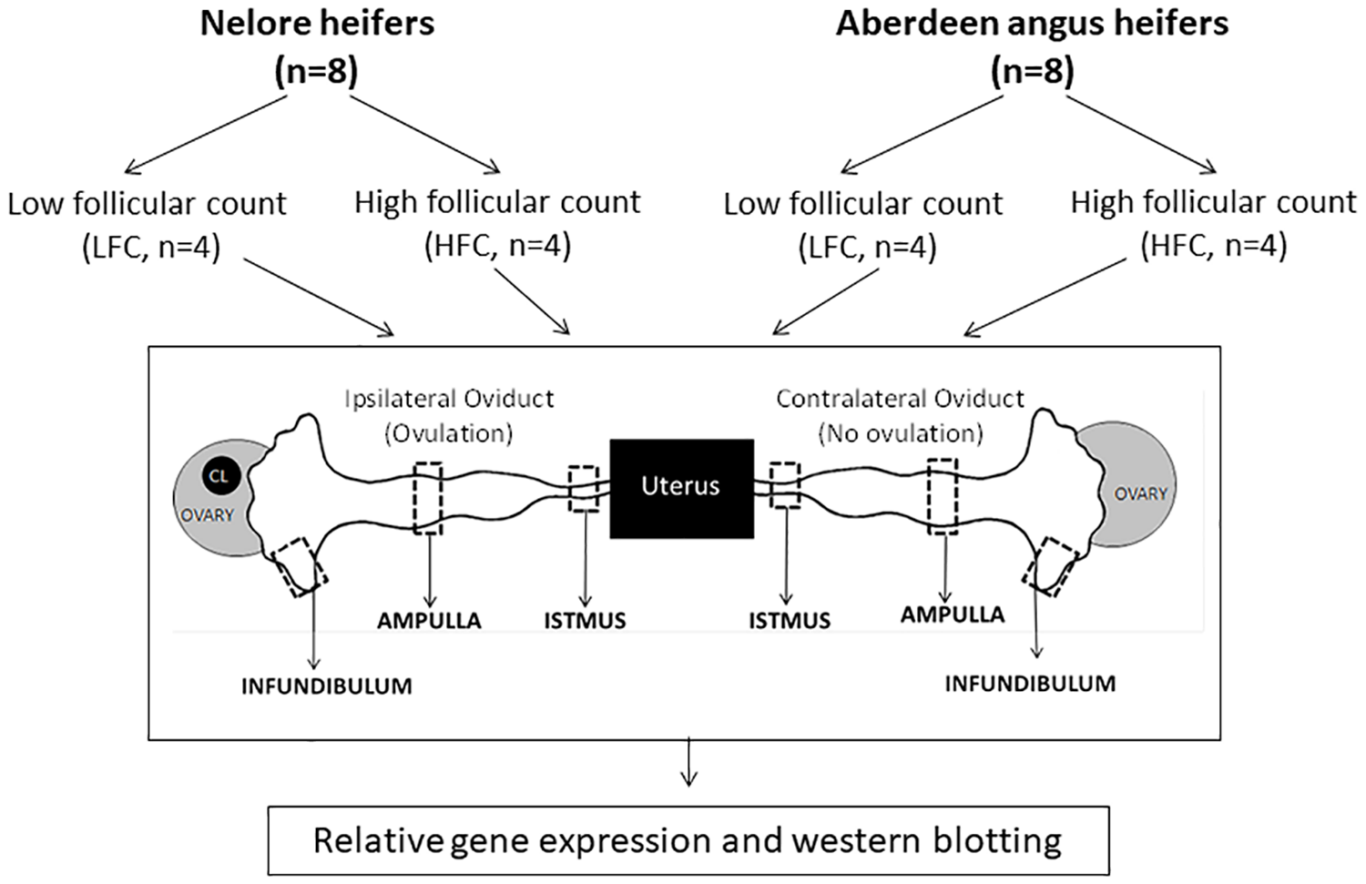
Experimental design. Aberdeen Angus (*Bos taurus taurus*, n = 8) and Nelore (*Bos taurus indicus*, n = 8) heifers were classified according to their ovarian AFC: low follicular count (LFC, n = 4) and high follicular (HFC, n = 4). All 16 animals were slaughtered one day after ovulation, and both oviducts were obtained. The ipsilateral and contralateral to ovulation side from each oviduct, including the infundibulum, ampulla, and isthmus segments, were submitted to relative gene expression by real time RT-PCR and protein quantification by western blotting.

For oviductal analysis, 16 heifers with an age of 24 months, a body condition score of 4 (0, emaciated; 5, obese), 345 kilograms (mean of Nelore heifers) and 330 kilograms (mean of Aberdeen Angus heifers) were used in the present study. The study analyzed eight Nelore heifers (n = 4/each AFC group) and eight Aberdeen Angus heifers (n = 4/each AFC group; Fig 1). The numbers of follicles were 15 ± 1 (LFC) and 53 ± 3 (HFC) in Nelore heifers, and 9 ± 2 (LFC) and 33 ± 2 (HFC) in Aberdeen Angus heifers (values presented as mean ± SD). All animals were studied simultaneously, at the same place and time while maintained on a pasture (*Brachiaria brizantha*), with *ad libitum* access to water. They were fed 2 kg of *Cynodon* spp. hay and 4 kg of concentrate (16% crude protein and 70% total digestible nutrients) per animal, per day for a total of 90 days.

### Sample collection

To synchronize the estrous cycle, the heifers were given two doses of prostaglandin F_2_a spaced over 11 days, and then the ovaries were evaluated by US every 12 hours until ovulation. The heifers were slaughtered in a commercial abattoir 24 hours after ovulation. One or two animals from each experimental group were slaughtered in each of the three independent sessions.

Blood samples were collected at the day of the slaughter, centrifuged (10 minutes at 900 x *g*), and the plasmatic antimullerian hormone (AMH) concentration was measured to confirm the phenotypes of the experimental animals [10]. Plasmatic AMH concentration in Nelore was higher (102.3 ± 6.4 pg/ml; p <0.001) when compared with Aberdeen Angus (78 ± 5.1 pg/ml) heifers. Moreover, the AMH concentration was higher (p <0.001) in heifers with HFC when compared to the LFC from Nelore (127 ± 8.8 and 84 ± 7.5 pg/ml) and Aberdeen Angus (82 ± 6 and 72 ± 6.9 pg/ml).

The reproductive tracts were then transported to the laboratory (approximately 2 hours of transportation) in saline solution (0.9%) at 4 °C. Ipsilateral and contralateral oviducts of the ovulation side of each animal were isolated, and the surrounding connective tissues were trimmed. The oviduct length was measured by a ruler, and then the oviducts were divided by segment: infundibulum, ampulla, and isthmus (the transition regions were discarded). Two fragments of each segment were collected and stored in −80 °C until gene and protein analysis (Fig 1).

### Sample preparation

Tissue samples (20 mg) were homogenized separately in CK28-R tubes (2 mL, with ceramics beads) by a Precellys^®^ homogenizer (Bertin Technologies^®^, Montigny le Bretonneux, France) after adding 500 μL lysis buffer, as follows: three cycles of 50 seconds at 6500 rpm with 15 second intervals. Total RNA and total protein were extracted using Illustra TriplePrep Kit (GE Healthcare, Buckinghamshire, UK), according to the manufacturer’s instructions.

### Real-time RT-PCR

Total RNA concentration was quantified by a spectrophotometer (Nanodrop 2000^™^, ThermoFisher Scientific, Wilmington, DE) and RNA quality was evaluated with a 2100 Bioanalyzer with RNA Nano chips (Agilent Technologies, Waldbronn, Germany). Samples of infundibulum had an RNA integrity number (RIN) > 7.0, ampulla > 7.5 and isthmus > 5.5.

Total RNA (1.2 μg) from each sample was incubated with DNAse I (Invitrogen^®^, CA, USA) and then reverse transcribed with a High Capacity cDNA kit (Applied Biosystems^™^, Carlsbad, CA), according to the manufacturer’s instructions. Relative RT-qPCR analysis was performed using TaqMan^®^ Low Density Array cards according to the manufacturer’s instructions (TLDA, Applied Biosystems^™^, Carlsbad, CA). The TLDA cards (384 wells) were designed with 24 genes in duplicate to analyze eight samples in each TLDA card (Table 1). Briefly, the TLDA card is a ready to use system, with selected TaqMan Gene Expression Assays pre-loaded into each of the 384 reactions (250 nM, final concentration 1 μL reaction volume). Individual samples were diluted with water to a final volume of 50 μL (total RNA load: 600 ng) and mixed with 50 μL TaqMan^®^ Universal PCR Master Mix (2X). Each mix (100 μL) was loaded in one of the eight channels of the TLDA card. The cards were sealed, spun, and submitted to standard PCR conditions: 50 °C for 2 minutes, followed by 95 °C for 1 minute, then 40 cycles of 95 °C for 15 seconds, and 60 °C for 1 minute in the ViiA7 PCR machine (Thermo Fisher Scientific). The intrassay variation CV for all PCR analysis was ≤ 15% of the cycle-threshold value.

**Table 1:** Genes analyzed in bovine oviducts using TLDA^®^ system (Gene symbols, gene full name, Applied Biosystems^™^ ID, function/reason for selection, and reference).

Gene	Gene full name	Taqman ID	Function/Reason for selection	Reference
*18S*	18S Ribosomal RNA	Hs99999901_s1	Reference gene	[33]
*ACE*	Angiotensin converting enzyme	Bt04300007_g1	Gamete transport	[34, 35]
*ACTB*	Beta-actin	Bt03279174_g1	Reference gene	[36]
*AGTR1*	Angiotensin II receptor, type 1	Bt03213473_m1	Gametes transport	[34, 35]
*ANXA1*	Annexin 1	Bt03224459_g1	Sperm reserve formation	[28]
*ANXA2*	Annexin 2	Bt03215891_g1	Sperm reserve formation	[28]
*ANXA4*	Annexin 4	Bt03210021_m1	Sperm reserve formation	[28]
*ANXA5*	Annexin 5	Bt03252080_g1	Sperm reserve formation	[28]
*ECE1*	Endothelin converting enzyme 1	Bt03217439_m1	Gamete transport	[37]
*EDN1*	Endothelin 1	Bt03217446_m1	Gamete transport	[37]
*FLT1*	VEGF receptor, type I receptor tyrosine kinase	Bt04302190_m1	Gamete transport	[35]
*FUCA1*	Fucosidase, α-L-1	Bt03238509_g1	Fertilization process; gamete interaction; control polyspermy	[31, 38]
*FUCA2*	Fucosidase, α-L-2	Bt04285945_m1	Fertilization process; gamete interaction; control polyspermy	[31, 38]
*GAPDH*	Glyceraldehyde-3-phosphate dehydrogenase	Bt03210913_g1	Reference gene	[36]
*HSPA5*	Heat shock protein family A (Hsp70) member 5	Bt03244880_m1	Fertilization process; gamete interaction; control polyspermy	[39, 40]
*KDR*	VEGF receptor, type III receptor tyrosine kinase	Bt03258885_m1	Gamete transport	[35]
*LHCGR*	Lutropin hormone receptor	Bt03213972_m1	Gamete transport	[21, 41]
*OVGP1*	Oviductal glycoprotein 1	Bt03253683_g1	Fertilization process; gamete interaction; control polyspermy	[14, 42]
*PPIA*	Peptidylprolyl isomerase A/Cyclophilin A	Bt03224615_g1	Reference gene	[36]
*PTGER2*	Prostaglandin E receptor 2	Bt03223848_m1	Gamete transport	[21, 35]
*PTGER4*	Prostaglandin E receptor 4	Bt03223849_m1	Gamete transport	[21, 35]
*PTGS1*	Prostaglandin-endoperoxidase synthase/Cyclooxygenase 1	Bt03817775_m1	Gamete transport	[21, 35]
*PTGS2*	Prostaglandin-endoperoxidase synthase/Cyclooxygenase 2	Bt03214492_m1	Gamete transport	[21, 35]
*VEGFA*	Vascular endothelial growth factor	Bt03213282_m1	Gamete transport	[35]

The relative expressions of target genes were calculated using 2(-ΔΔCt) [43]. To select the most stable reference gene for oviduct analysis, the gene expression, amplification profiles, and Ct Values of peptidylprolyl isomerase A (*PPIA*), beta-actin (*ACTB*), glyceraldehyde-3-phosphate dehydrogenase (*GAPDH*), and 18S ribosomal RNA (*18S*) were tested among the different experimental groups and compared using the GeNorm applet [44] for RefFinder web-based software (http://leonxie.esy.es/RefFinder/) [45]. The most stable references genes were *PPIA* and *18S*.

### Western blotting

Total protein quantification was performed by the Bradford method in 96 well plates, and the samples were analyzed by a spectrophotometer at 595 nm. Aliquots (70 μg of protein) were treated with a buffer solution (Laemmli sample buffer BIO-RAD) and beta-mercaptoethanol at 100 °C for 5 minutes. The proteins were then separated by SDS-PAGE and transferred to a nitrocellulose membrane. Nonspecific binding of proteins was blocked by incubating the membrane in 5% skim milk-TBST buffer for 1 hour at room temperature. The membranes were incubated with primary antibody FUCA-1 (rabbit polyclonal ab-98310, 1:1000, Abcam Inc., Cambridge, MA) or GAPDH (rabbit polyclonal sc47724, 1:1000, Santa Cruz Biotechnology VR, Inc., Dallas, TX) in 5% skim milk in TBST at 4 °C overnight.

After washing four times in TBST, membranes were incubated with specific HRP secondary antibody (IgG goat-anti rabbit, ab97051, 1:20,000, Abcam Inc.) in 5% skim milk in TBST for 2 hours at room temperature. After washing four times in TBST, immunoreactive components were visualized by chemiluminescence (ELC Select TM Western Blotting Detection Reagent, GE Healthcare^®^, UK).

Protein expression was tested in a subset of samples (total of 8 animals), as a pre-analysis of transcript genes indicated minimal effect concerning breed and AFC. The protein abundance was determined by semi-quantitative assays through band densitometry using Image J software (version 1.33u, National Institutes of Health, USA), normalized by GAPDH density. The integrated optical density (IOD) of the band was used as the unit of measure; mean and standard error of the mean (SEM) of IODs were compared among the groups and submitted to statistical tests.

### Statistical analysis

For all analyses, except for total oviduct length measurement, each oviduct segment was evaluated separately. Oviduct lengths were transformed to logarithmic values for a normal distribution and then tested by ANOVA (the cows were divided into two groups: Nelore and Aberdeen Angus, as there was no influence of AFC for this analysis). For gene expression analysis, each target gene was normalized using the geometric mean of two reference genes (*18S* and *PPIA*) and one calibration sample by 2(-ΔΔCt); [43]. Gene expression values were analyzed to determine the effect of triple interactions (breed vs. AFC vs. ovulation side; n = 4 animals/group), double interactions (breed vs. AFC, breed vs. ovulation side, and AFC vs. ovulation side; n = 4 animals/group), and main effects (breed, AFC, and ovulation side; n = 8 animals/group). Analysis of the ovulation side (ipsilateral and contralateral) considered them as dependent samples, since they come from the same animal. The responses were estimated by fitting linear mixed models after transforming the responses to a logarithmic scale. Multiple comparisons were performed by using the Bonferroni correction of *p*-values.

The total protein quantification by Bradford assay was analyzed to determine the effect of breed, AFC, and ovulation site. The responses were estimated by fitting linear mixed models after transforming the responses to a logarithmic scale. Multiple comparisons were performed by using the Bonferroni correction of *p*-values. Each oviductal segment was analyzed separately. For protein quantification by western blotting, each oviductal segment was analyzed separately, and only the difference in ovulation side (ipsilateral and contralateral) was evaluated for protein abundance. Relative abundance of FUCA1/GAPDH was estimated using Image-J, and then the data were transformed to the logarithmic for a normal distribution and tested with a Student’s t-test.

All analysis was performed using Proc mixed, SAS version 9.3 (SAS, 2010–2015). The differences were considered significant when *p* ≤ 0.05, and data are represented by mean ± SEM.

## Results

### Oviductal length

**Fig 2:**
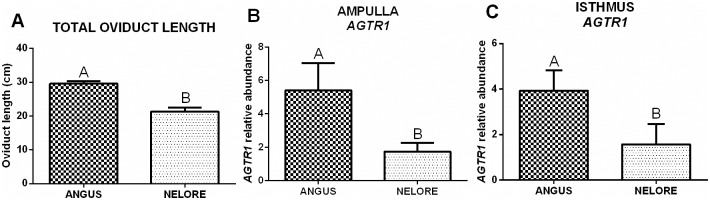
Oviductal differences between bovine breeds: Aberdeen Angus (n = 8 animals) vs. Nelore (n = 8 animals). (A) Total length of oviducts from each breed (mean ± SEM, centimeters). (B,C) Relative mRNA abundance (mean ± SEM) of *AGTR1* in the ampulla (B) and in the isthmus (C) from each breed. The target gene was normalized by reference genes using 2(-ΔΔCt). Different letters indicate significant difference (*p* ≤ 0.05) between means.

The total length of oviducts from Aberdeen Angus heifers (29.6 ± 0.7 cm; n = 8 animals) was longer than those from Nelore heifers (21.4 ± 1.8 cm; n = 8 animals, *p* < 0.0001; Fig 2A). No significant effects of ovulation side (ipsilateral *vs*. contralateral) or AFC (LFC *vs*. HFC) were found in this parameter (data not shown).

### Gene expression

All target genes were detected in the three bovine oviductal segments, except for the *LHCGR* transcript, which was not detected in half of the samples and impaired the analysis of its expression. Furthermore, not all genes were affected by differences in heifer breeds, AFC, or ovulation side (Table 2). In general, gene expression analysis was not affected by triple interaction (breed vs. AFC vs. ovulation side) and the isolated AFC characteristic. The most constitutive effect on the transcriptional profile of oviducts is caused by the ovulation side. Regarding isolated breed effects, only mRNA abundance of *AGTR1* was higher in Aberdeen Angus when compared to Nelore in the ampulla and isthmus segments (Fig 2B and 2C).

**Table 2:** *p* values for transcript abundance in each oviductal segment.

Gene symbol	Breed* AFC*side	Breed* AFC	Breed* side	AFC* Side	Breed	AFC	Side
**Infundibulum**							
*ACE*	0,39	0,86	0,17	**<0,01**	0,72	0,72	0,58
*AGTR1*	0,92	0,65	0,79	0,92	0,59	0,88	0,55
*ANXA1*	0,29	0,89	0,20	0,17	0,90	0,82	0,08
*ANXA2*	0,33	0,89	0,68	0,45	0,89	0,63	**0,05**
*ANXA4*	0,22	0,94	0,09	0,15	0,73	0,66	0,09
*ANXA5*	0,91	0,40	0,91	0,07	0,61	1,00	0,99
*ECE1*	0,45	0,82	0,21	0,16	0,35	0,40	0,09
*END1*	0,57	0,45	0,49	0,19	0,44	0,60	0,09
*FLT1*	0,49	0,92	0,34	0,30	0,19	0,59	0,22
*FUCA1*	0,54	0,38	**0,02**	0,37	0,24	0,08	0,02
*FUCA2*	0,12	0,81	0,98	0,82	0,57	0,53	**0,01**
*HSPA5*	0,44	0,42	0,76	0,50	0,96	0,66	**0,04**
*KDR*	0,22	0,95	0,80	0,98	0,84	0,64	0,39
*OVGP1*	0,97	0,37	**0,05**	0,16	0,89	0,10	0,06
*PGTER2*	0,47	0,86	0,45	0,17	0,95	0,98	0,13
*PGTER4*	0,20	0,80	0,95	0,48	0,24	0,12	**0,03**
*PTGS1*	0,35	0,51	0,84	0,20	0,95	0,46	0,10
*PTGS2*	0,20	0,84	0,79	0,62	0,33	0,74	0,28
*VEGFA*	0,55	0,76	0,85	0,15	0,85	0,77	**0,05**
**Ampulla**							
*ACE*	0,81	0,63	0,76	0,24	0,07	0,19	0,50
*AGTR1*	0,54	0,77	0,33	0,29	**0,02**	0,36	0,11
*ANXA1*	0,56	0,48	0,29	0,73	0,66	0,75	0,19
*ANXA2*	0,55	0,63	0,29	0,16	0,28	0,24	0,45
*ANXA4*	0,74	0,48	0,34	0,52	0,38	0,36	**0,04**
*ANXA5*	1,00	0,37	0,41	0,54	0,67	0,09	0,11
*ECE1*	0,62	0,46	0,52	0,56	0,38	0,69	0,11
*END1*	0,80	0,69	0,26	0,87	0,49	0,45	**0,05**
*FLT1*	0,07	0,35	0,45	0,62	0,82	0,87	0,35
*FUCA1*	0,17	0,85	0,89	**<0,01**	0,26	0,89	0,04
*FUCA2*	0,86	0,75	0,17	0,31	0,59	0,28	0,41
*HSPA5*	0,75	0,29	0,57	0,51	0,72	0,36	**0,03**
*KDR*	0,46	0,84	0,74	0,12	0,33	0,39	0,35
*OVGP1*	0,26	0,79	0,74	0,97	0,20	0,60	**0,02**
*PGTER2*	0,90	0,65	0,32	0,22	0,49	0,38	0,22
*PGTER4*	0,68	0,45	0,67	0,37	0,29	0,42	0,07
*PTGS1*	0,74	0,37	0,54	0,81	0,31	0,40	0,25
*PTGS2*	0,80	0,32	0,85	0,84	0,20	0,46	**0,01**
*VEGFA*	0,94	0,60	0,67	0,52	0,75	0,26	0,12
**Isthmus**							
*ACE*	0,99	0,39	0,43	0,36	0,11	0,96	0,30
*AGTR1*	0,87	0,28	0,40	0,07	**0,05**	0,64	0,14
*ANXA1*	0,75	0,62	0,27	0,58	0,81	0,28	0,64
*ANXA2*	0,37	0,96	0,43	0,44	0,96	0,19	0,08
*ANXA4*	0,67	0,68	0,26	0,76	0,97	0,10	0,58
*ANXA5*	0,73	0,23	0,98	0,73	0,78	0,78	0,13
*ECE1*	0,99	0,98	0,42	0,53	0,95	0,12	0,40
*END1*	0,63	0,44	0,82	0,11	0,96	0,95	0,46
*FLT1*	0,66	0,88	0,26	0,55	0,58	0,10	0,72
*FUCA1*	0,49	0,92	0,11	0,77	0,99	0,25	0,66
*FUCA2*	0,94	0,99	0,37	0,35	0,75	0,12	**0,03**
*HSPA5*	0,93	0,96	0,19	0,27	0,86	0,09	0,97
*KDR*	0,42	0,62	0,83	0,70	0,91	0,91	**0,02**
*OVGP1*	0,35	1,00	0,98	0,16	0,95	0,22	0,51
*PGTER2*	0,70	0,38	0,35	0,81	0,65	0,28	0,27
*PGTER4*	0,34	0,98	0,11	0,81	0,49	0,40	0,27
*PTGS1*	0,66	0,23	0,82	0,14	0,91	0,81	0,68
*PTGS2*	0,68	0,82	0,35	0,53	0,21	0,40	0,51
*VEGFA*	0,32	0,61	0,42	**0,05**	0,82	0,50	0,03

The interactions of three effects were analyzed: breed *vs*. AFC *vs*. side; two effects: breed *vs*. AFC, breed *vs*. side, AFC *vs*. side; and individual effects: breed (Aberdeen Angus *vs*. Nelore), AFC (LFC *vs*. HFC), and the ovulation side (ipsilateral *vs*. contralateral).

**Fig 3:**
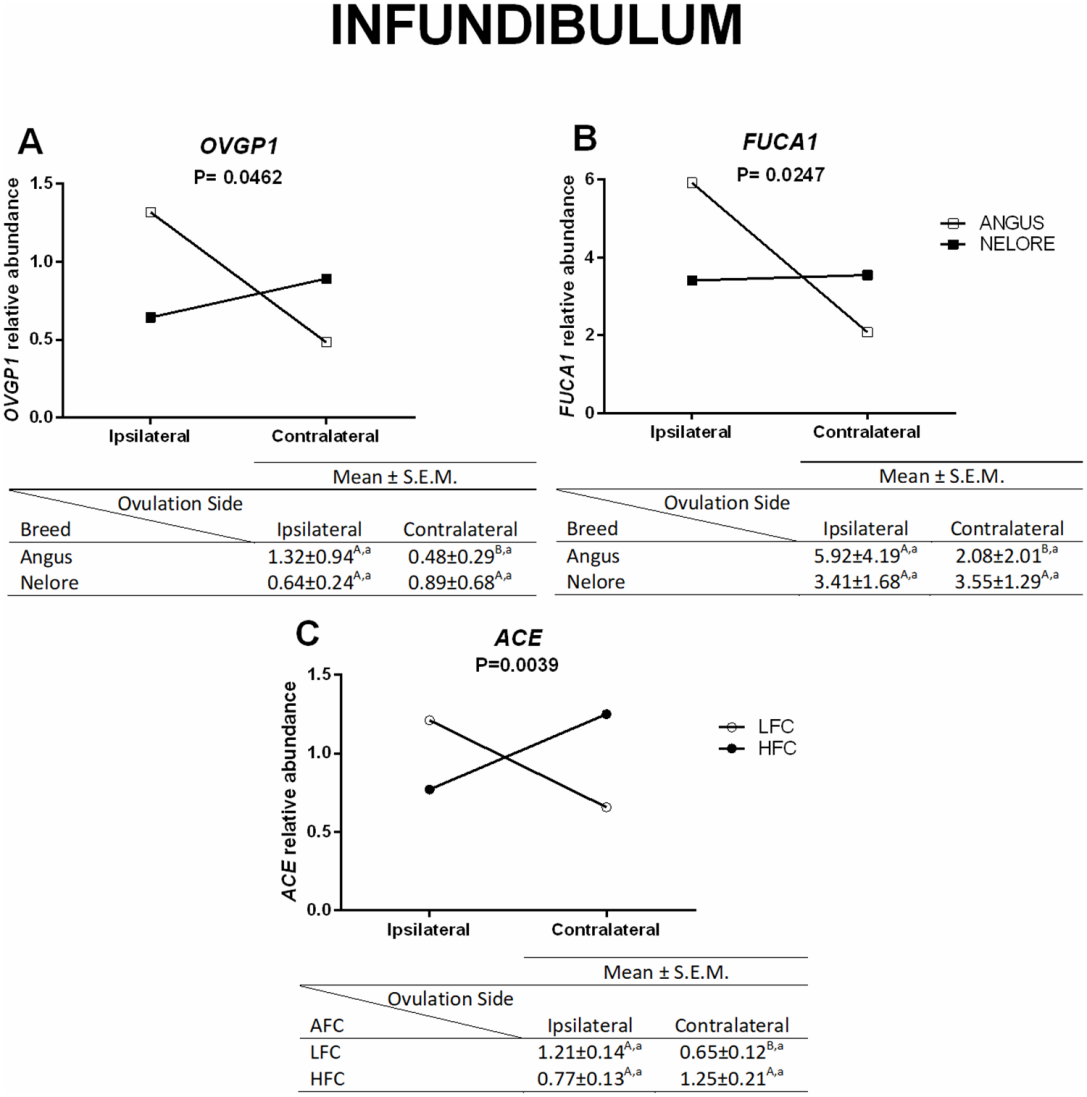
Differences in transcripts levels in the infundibulum (ovulation side/breed and ovulation side/AFC). Relative mRNA abundance (mean ± SEM) of *OVGP1* (A) and *FUCA1* (B) in ipsilateral and contralateral oviducts from Aberdeen Angus (n = 8 animals) and Nelore (n = 8 animals) heifers were normalized by reference genes using 2(-ΔΔCt). The analyses were performed by comparing different ovulation sides in the same breed and the same ovulation side in different breeds, as well as relative mRNA abundance of *ACE* (C) in ipsilateral and contralateral oviducts from LFC (n = 8 animals) and HFC (n = 8 animals) heifers. The analyses were performed by comparing different ovulation sides in the same AFC groups, and the same ovulation sides in different AFC groups. Different uppercase letters (^A,B^) indicate significant differences (*p* ≤ 0.05) between ovulation sides in the same breed/AFC group (horizontal analysis). Different lowercase letters (^a,b^) indicate significant difference *(p ≤* 0.05) between breed/AFC of same ovulation side (vertical analysis).

**Fig 4:**
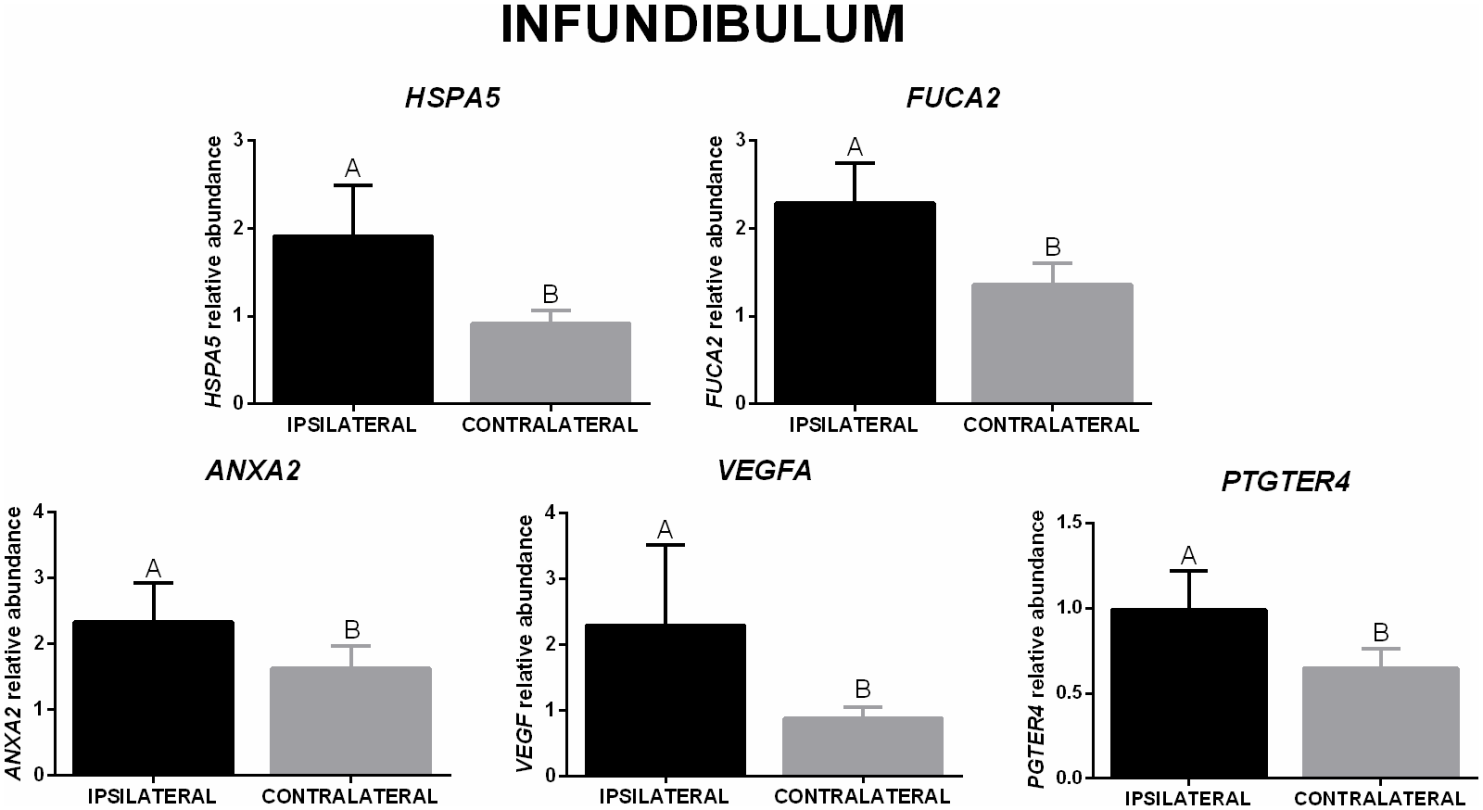
Relative mRNA abundance in the infundibulum (ovulation side effect). *HSPA5*, *FUCA2*, *ANXA2*, *VEGFA*, and *PTGER4* (mean ± SEM) in ipsilateral and contralateral oviducts (n = 16 animals). Expression of target genes were normalized by reference genes using 2(-ΔΔCt). Different letters indicate significant difference *(p* ≤ 0.05) between means.

In the infundibulum, higher mRNA abundance of *OVGP1* and *FUCA1* was present in the ipsilateral when compared to the contralateral oviduct from Aberdeen Angus. A similar effect was not present in Nelore heifers, and there was no significant difference between breeds (Fig 3A and 3B). Moreover, the relative abundance of *ACE* changed based on the side of the LFC heifers (higher levels in the ipsilateral compared to contralateral), but not in the HFC (Fig 3C). When only comparing the ovulation side in the infundibulum, a higher mRNA abundance of *HSPA5*, *FUCA2*, *ANXA2*, *VEGFA*, and *PTGER4* was present in the ipsilateral oviduct when compared to the contralateral (Fig 4).

**Fig 5:**
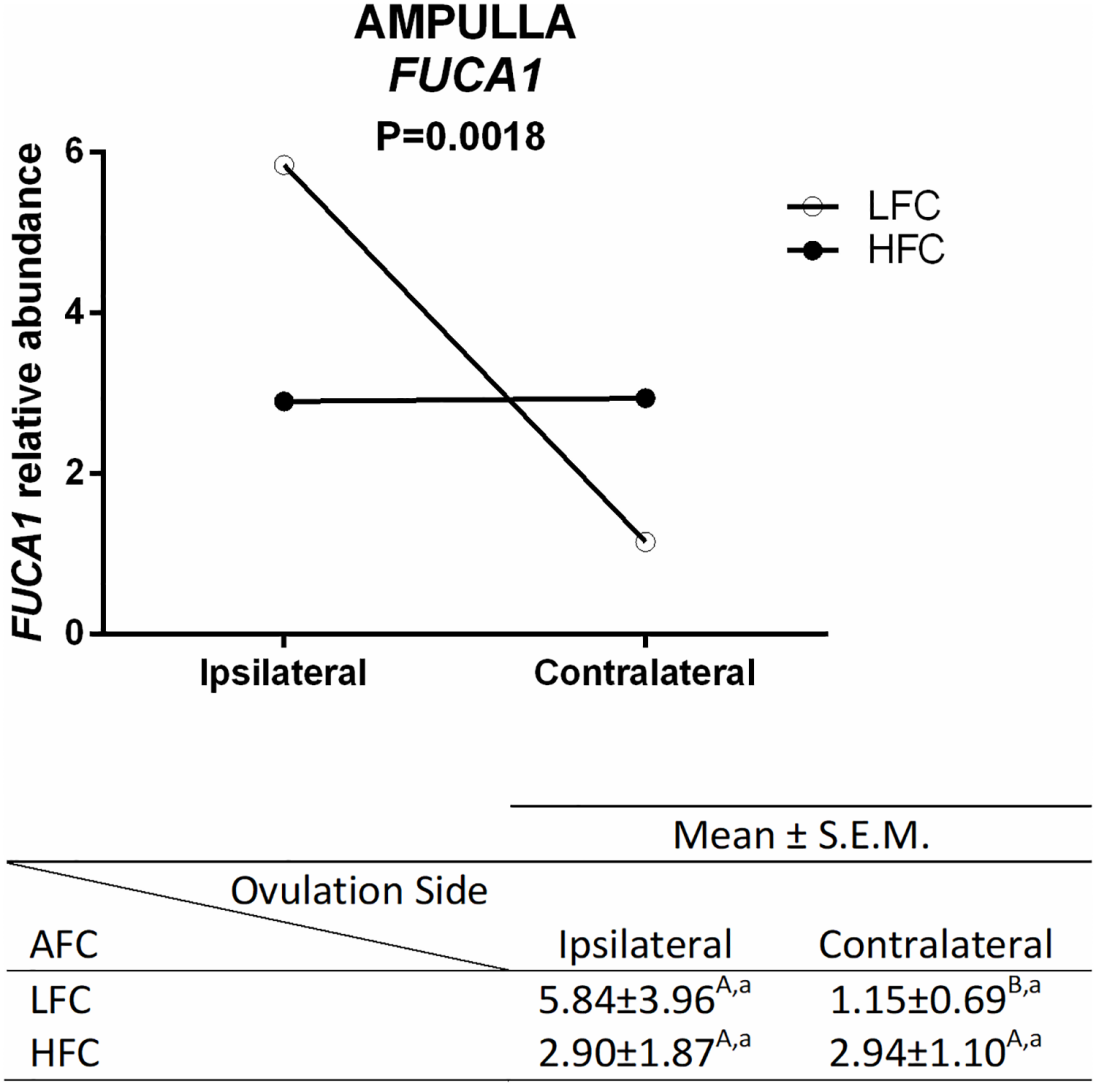
Relative mRNA abundance of *FUCA1* in ipsilateral and contralateral ampulla from LFC (n = 8 animals) and HFC (n = 8 animals) heifers. Expression of the target gene was normalized by references genes using 2(-ΔΔCt), and relative abundance is presented as mean ± SEM. The analyses were performed by comparing different ovulation sides in the same AFC groups, and the same ovulation side in different AFC groups. Different uppercase letters (^A,B^) indicate significant difference (*p* ≤ 0.05) between ovulation sides in the same AFC group (horizontal analysis). Different lowercase letters (^a,b^) indicate significant difference *(p* ≤ 0.05) between AFC groups on the same ovulation side (vertical analysis).

**Fig 6:**
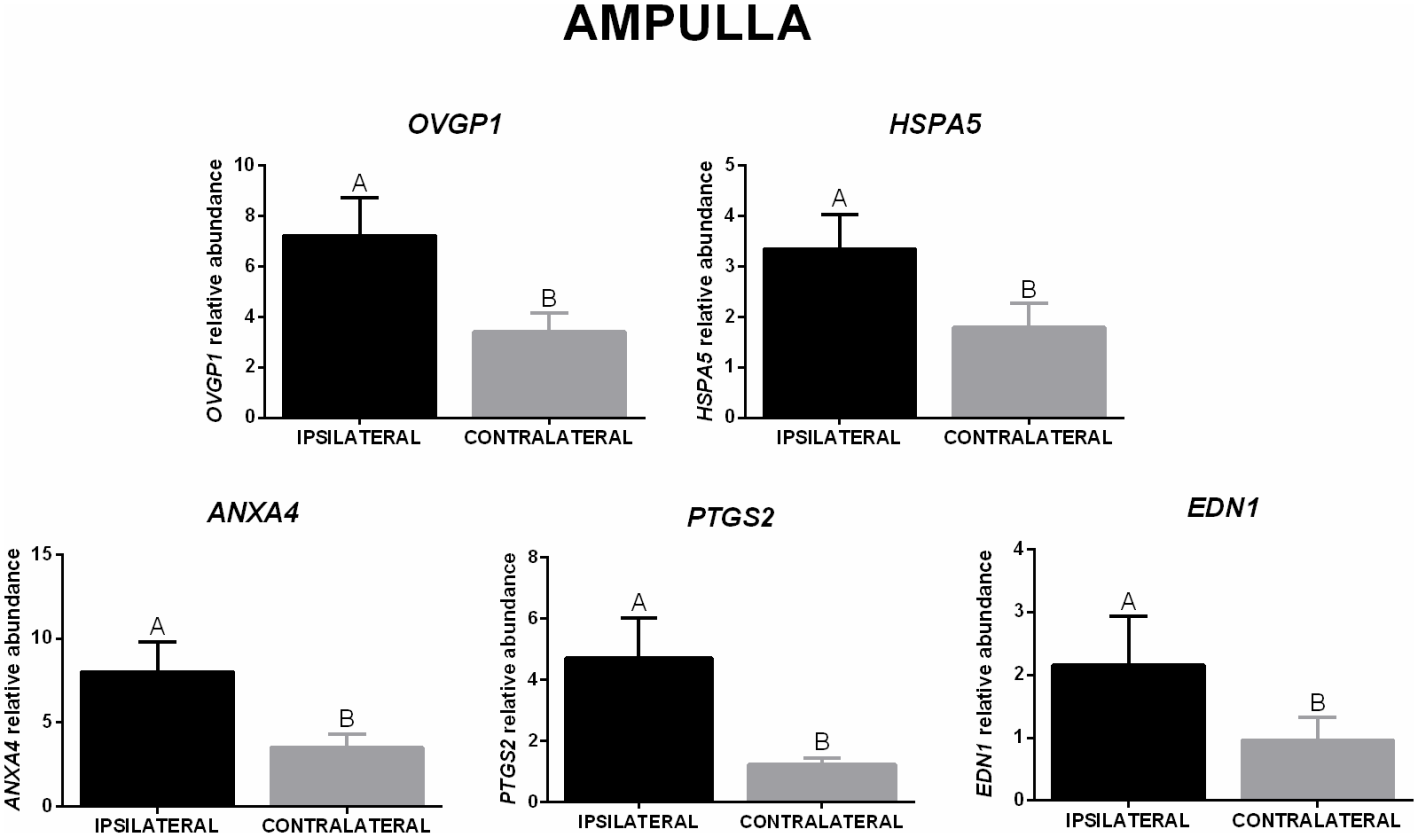
Transcript levels in the ampulla (ovulation side effect). Relative mRNA abundance (mean ± SEM) of *OVGP1*, *HSPA5*, *ANXA4*, *PTGS2*, and *END1* in ipsilateral and contralateral oviducts (n = 16 animals) were normalized by reference genes using 2(-ΔΔCt). Different letters indicate significant difference *(p* ≤ 0.05) between means.

In the ampulla, mRNA abundance of *FUCA1* was higher in the ipsilateral than in the contralateral oviducts in LFC heifers, but this effect was not detected in the HFC heifers, and there was no significant difference between AFC groups in the same ovulation side (Fig 5). Regarding ovulation side, mRNA abundance of five genes was affected; *OVGP1*, *HSPA5*, *ANXA4*, *PTGS2*, and *END1* mRNA were higher in ipsilateral ampulla when compared with the contralateral (Fig 6).

**Fig 7:**
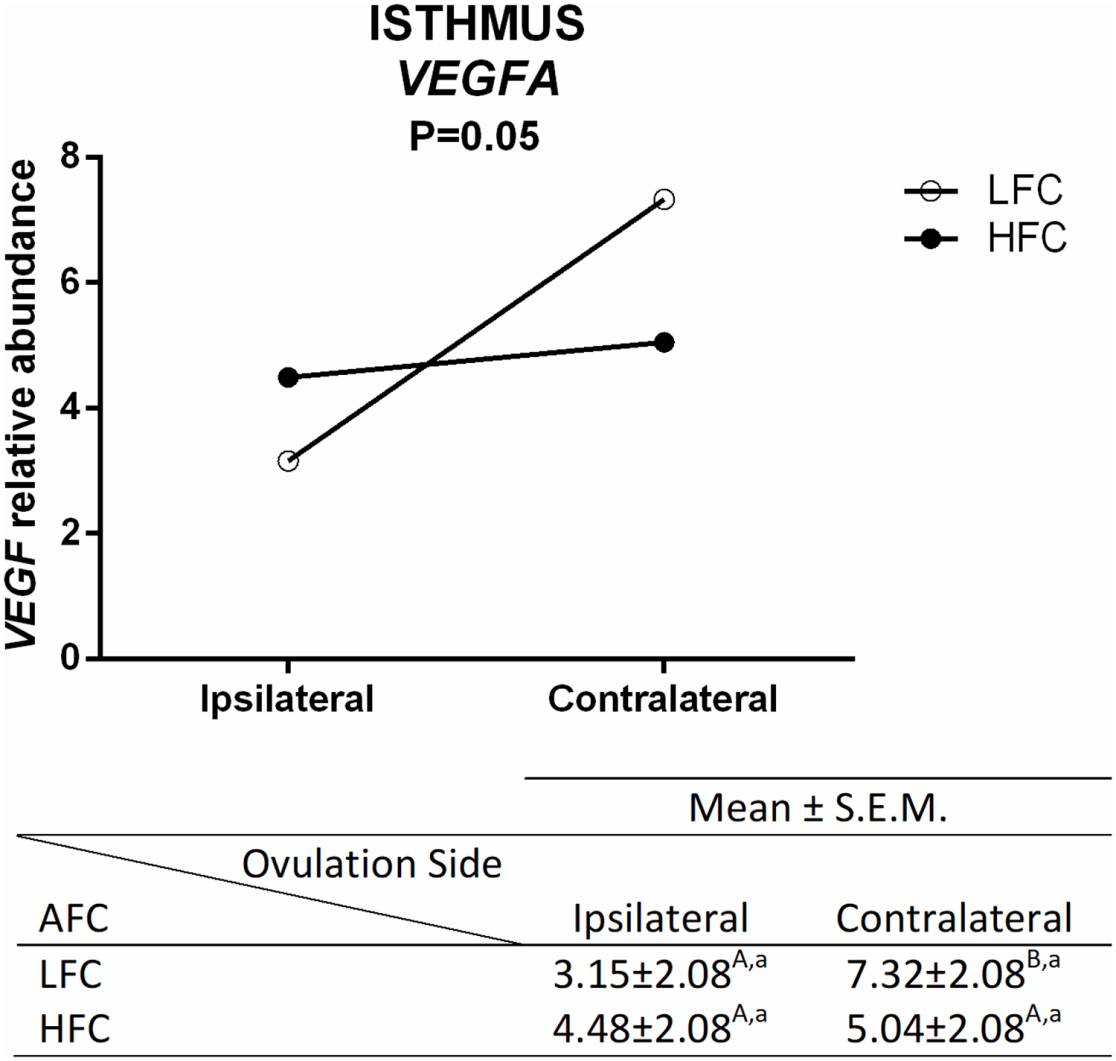
Transcript levels in the isthmus were different depending on ovulation side and AFC. Relative abundance (mean ± SEM) of *VEGFA* in ipsilateral and contralateral oviducts from LFC (n = 8 animals) and HFC (n = 8 animals) heifers were normalized by reference genes using 2(-ΔΔCt). The analyses were performed by comparing different ovulation sides in the same AFC group, and the same ovulation side in different AFC groups. Different uppercase letters (^A,B^) indicate significant difference (*p* ≤ 0.05) between ovulation sides in the same AFC (horizontal analysis). Different lowercase letters (^a,b^) indicate significant difference *(p ≤* 0.05) between AFC groups on the same ovulation side (vertical analysis).

**Fig 8:**
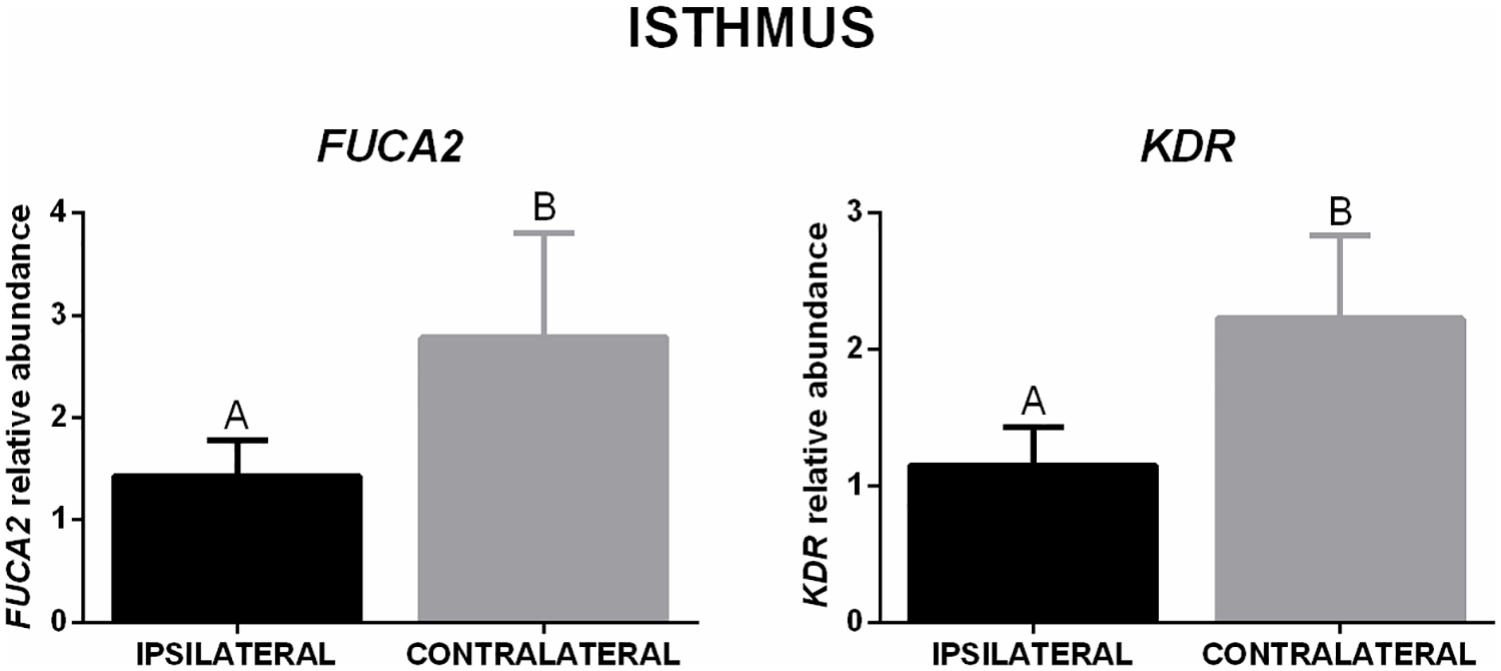
Transcript levels in the isthmus (ovulation side effect). Relative mRNA abundance (mean ± SEM) of *FUCA2* and *KDR* in ipsilateral and contralateral oviducts (n = 16 animals). Target genes were normalized by reference genes using 2(-ΔΔCt). Different letters indicate significant difference (*p* ≤ 0.05) between means.

In the isthmus, a lower number of genes was affected. The mRNA abundance of *VEGFA* was lower in ipsilateral when compared to the contralateral isthmus from LFC heifers, but no difference was detected in the HFC animals, and there was no difference between different AFC groups in the same ovulation side (Fig 7). Regarding the difference in the ovulation side, mRNA abundance of *FUCA2* and *KDR* was lower in the ipsilateral isthmus when compared to the contralateral (Fig 8).

### Protein abundance

**Fig 9:**
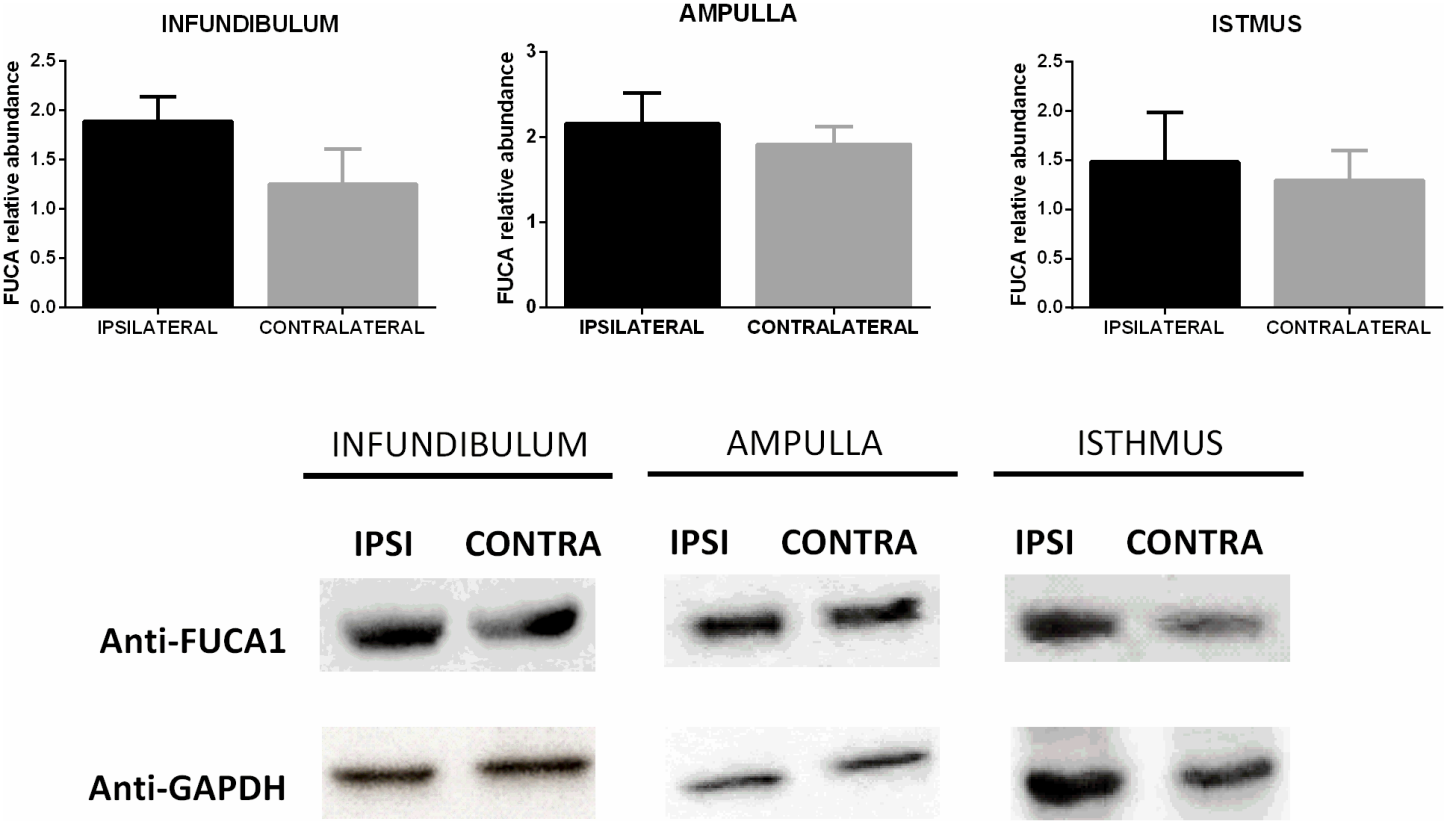
Protein levels of FUCA1 in the infundibulum, ampulla, and isthmus. Relative protein abundance (FUCA1/GAPDH, mean ± SEM) in the ipsilateral and contralateral oviducts (n = 8 animals), *p* > 0.05.

The cattle breed, AFC, and ovulation side had no effect on the total protein levels in the infundibulum and isthmus. However, in the ampulla, the AFC had a significant effect (*p* < 0.01) with greater levels in the LFC (8.99 ± 0.68 μg/μL) than the HFC (5.70 ± 0.84 μg/μL). When comparing the ipsilateral and the contralateral oviducts (Fig 9), the relative abundance of FUCA1 in the infundibulum, ampulla, and isthmus was similar.

## Discussion

To our knowledge, for the first time, the impact in variation of the antral follicle count (AFC) on oviductal gene expression was evaluated in two cattle breeds used for beef production. Genes reported as potentially responsible for oviductal transport, sperm reservoir formation, monospermy control, and gamete interaction presented a minimal difference in the oviducts comparing animals with high or low AFC, independent of the cattle breed. However, a clear interaction between AFC and the oviduct side indicates that HFC animals have similar gene abundance in both oviducts, while the abundance of some genes in LFC animals is very different when the ipsilateral and contralateral are compared.

After the phenotypic classification of heifers based on variation in AFC (low vs. high), several studies have tried to determine the correlation of AFC increased fertility in cattle. It is clear that there are benefits for HFC animals when compared to LFC regarding reproductive biotechnology, including ovarian superovulation and *in vitro* embryo production, as the total number of structures (oocytes and embryos) is higher in HFC animals [46]. However, the efficiency of embryo production is controversial, some studies observed a similar result between animals with different AFC [1, 47], better in HFC animals [2], or better in LFC animals [46]. Other studies have demonstrated normal sizes of corpus luteum and endometrial thickness between AFC groups [4], while other studies have showed poor endometrial development [4] and lower protein content in the uterus of LFC animals [11]. These findings suggest AFC variation is not a clear factor in bovine fertility modulation.

In this present work, we demonstrated an interaction between AFC and the ovulation side regarding the mRNA abundance of some genes. The HFC animals have no difference between ovulation sides; however, in LFC animals, some factor may regulate differential abundance of *ACE* (in the infundibulum), *FUCA1* (in the ampulla), and *VEGFA* (in the isthmus) between ipsi- and contralateral oviducts. Moreover, LFC animals presented a higher total protein concentration in the ampulla when compared to HFC animals. Taken together, these findings might not solve the discussion about the relationship of AFC and bovine fertility, but we suggest that there is no detrimental effect in the oviduct of animals with LFC.

There are some physiological differences between breeds, e.g., estrous cycle length, size of pre-ovulatory follicle, and steroid hormone concentration (estradiol and progesterone [48–52]). In the present study, the total length of Aberdeen Angus (*Bos taurus taurus*) oviducts were longer than those of Nelore (*Bos taurus indicus*) heifers. We theorized that this difference in length suggests that gametes and embryos from Aberdeen Angus have a longer course during transport in the oviduct compared to Nelore heifers, and perhaps some compensatory mechanism is necessary to guarantee that the gametes and embryos are transported on time. Indeed, mRNA abundance of *AGTR1* in the ampulla and isthmus from the oviducts of Aberdeen Angus was higher than that of Nelore heifers. Angiotensin II (AGTII) participates in reproductive physiology [53] by interacting with at least two receptor subtypes—AGTR1 and AGTR2 [54]. The AGTR1 receptor mediates effects of AGTII in the oviduct to stimulate smooth muscle contraction [34] and CBF [25]. Therefore, the upregulation of *AGTR1*, in the present study, may cause a stimulatory effect on oviductal CBF and faster transport in the oviducts of Aberdeen Angus heifers, making AGTII a potential factor in the compensatory mechanism of this breed.

**Fig 10:**
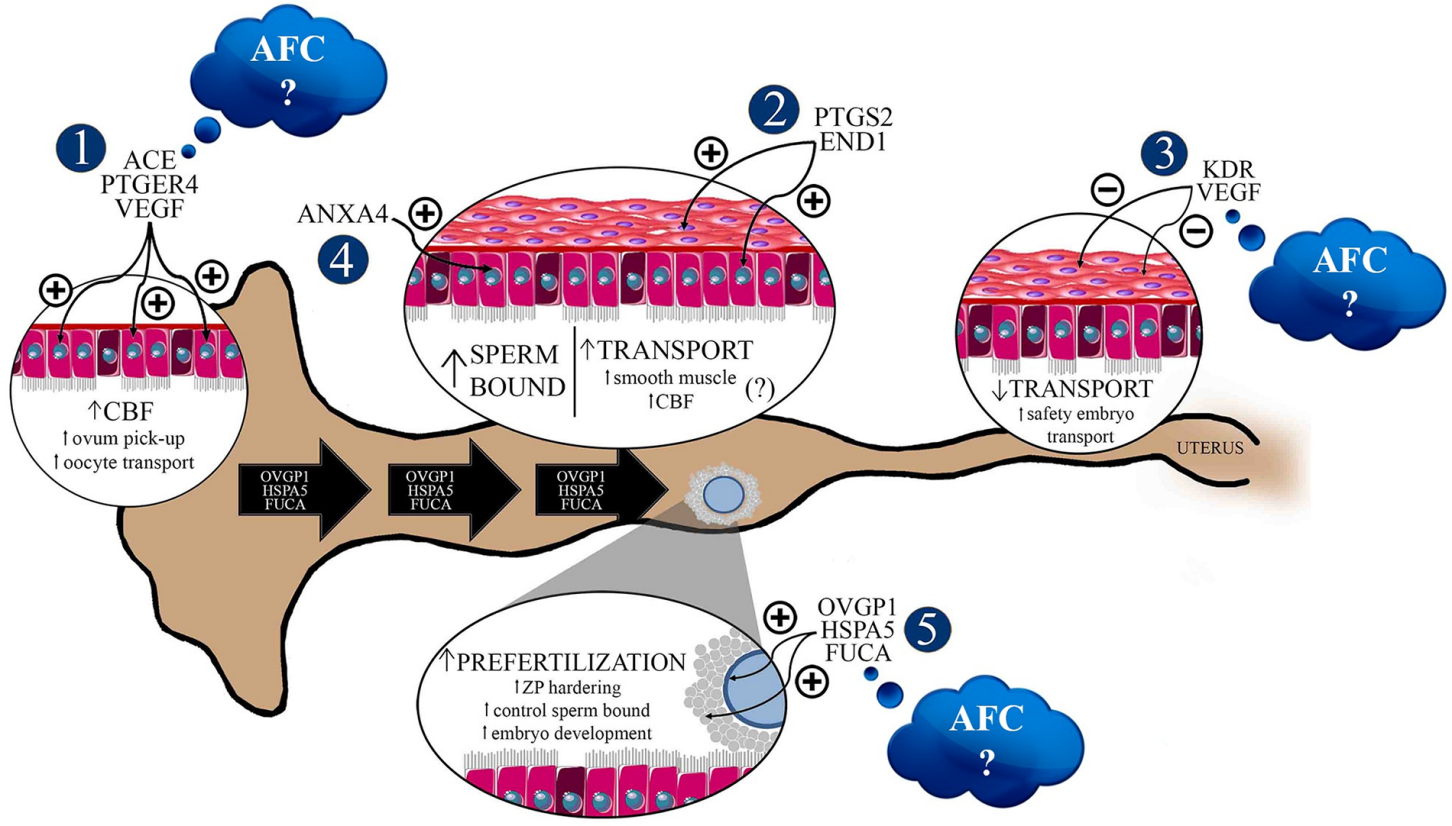
Biological status of bovine ipsilateral oviducts, one day after ovulation, in Aberdeen Angus or Nelore heifers. The ovulation side was the major factor that modulates the molecular profiles of bovine oviducts. There is a possibility of modification in the ipsilateral oviduct microenvironment to control gamete transportation, polyspermy gamete interaction, and embryo development even without the occurrence of mating, sperm presence, or fertilization. These oviductal functions could be controlled by ovulation products. 1. Upregulation of VEGF, PGE2, and ANGII systems in the infundibulum increase CBF, resulting in efficient ovum pick-up and oocyte transportation to the fertilization site. 2. Upregulation of PTGs and END1 systems in the ampulla possibly raises oviductal transport by stimulatory effects on smooth muscle contraction or CBF. 3. Downregulation of the VEGF system in the isthmus, caused by negative feedback, guarantees slow and safe transport of the embryo to the uterus. 4. Upregulation of annexin transcripts in the ampulla possibly results in an increase of sperm-bound sites, thereby controlling sperm release to oocyte fertilization. 5. Upregulation of *OVGP1*, *HSPA5*, and *FUCA* levels from the infundibulum to the ampulla may increase the activity of these factors on the oocytes before fertilization, participating in ZP hardening, control of sperm bound to ZP, and embryo development. We observed a difference between ipsi- and contralateral oviducts concerning the abundance of ACE (in the infundibulum), FUCA (in the ampulla), and VEGFA (in the isthmus) from low-AFC cows, but they were similar in both sides of the oviducts in high-AFC cows. The modulation by the AFC is still inconclusive, and more experiments should be performed to confirm if this subtle effect of AFC on oviduct functions can impact animal reproduction. AFC: antral follicle count, CBF: ciliary beat frequency, ZP: Zona Pellucida; CL: Corpus Luteum; (−) downregulated factor, (+) upregulated factor. Black large arrows represent the *OVGP1*, *HSPA5*, and *FUCA* coming from the infundibulum and ampulla.

Despite this, we observed that the major modulations in genes related to oviductal functions were associated with ovulation side. Fertilization and embryo initial development are regulated by oviductal fluid (OF) production, and the identification of specific proteins from OF is important in the understanding of physiology and for the application of this knowledge to reproduction biotechnology. In the present study, the ovulation side modulated the levels of *OVGP1* (infundibulum from Angus heifers), *OVGP1* (ampulla from all animals), and *HSPA5* (infundibulum and ampulla from all animals) transcripts; they were higher in the ipsilateral compared to the contralateral oviduct, suggesting an increased functional activity of these proteins in ipsilateral segments. Oviductal glycoprotein 1 (OVGP1) is a protein present in the oviduct related to zona pellucida (ZP) solubility modifications, and consequently enhances sperm penetration resistance [31, 42] and leads to monospermy [42, 55]. Heat shock 70 kDa protein 5 (HSPA5, previously known as Glucose-Regulated Protein, 78 kDa, GRP78) interacts with sperm, improving their viability, acrosomal integrity, and sperm movement [39, 56, 57]. HSPA5 also modulates sperm-ZP interactions [58] and possibly participates in ZP hardening mechanisms, regulating monospermy levels [31]. Therefore, current data suggests that upregulation of *OVGP1* and *HSPA5* in ipsilateral oviducts could be involved in the prevention of polyspermy by modulating ZP hardening, even before fertilization [31] (Fig 10).

FUCA is an acidic glycosidase that catalyzes the hydrolytic degradation of fucose [38]. It is present in the reproductive system [59] and involved in different roles mediated by OF. In cattle, there are two types of fucosidases, FUCA1 (α-L-1-fucosidase) and FUCA2 (α-L-2-fucosidase), and the difference in function of each protein in the oviduct is not yet understood. In the present study, both *FUCA1* and *FUCA2* were detected in all oviduct segments. In the isthmus, sperm enters the oviduct and binds to ciliary epithelial cells—more specifically to fucose residues—for reservoir formation [29]. FUCA present in the OF regulates sperm release from the isthmic reservoir, controlling the number of sperm reaching the fertilization site [29], which is increased after ovulation [60]. Surprisingly, in the present study, *FUCA2* abundance was lower in the ipsilateral isthmus compared to the contralateral side, one day after ovulation. It is possible that sperm interaction is more involved in isthmus regulation. Several studies have showed the importance of sperm in isthmus modulation, and confirmed a cross-talk between gamete and oviductal cells [61–66]. The absence of sperm in the experimental setup of the present study might be responsible for the downregulation of genes in the ipsilateral isthmus region (Fig 10).

Previous studies showed the participation of fucosidase during fertilization and embryo development. Pre-incubation of oocytes with FUCA decreased the number of sperm bound to ZP [67], while the inhibition of FUCA activity reduced sperm penetration during bovine *in vitro* fertilization [68, 69], and in turn, the oocyte was unable to pass the 2-pronuclear stage [69]. In the present study, higher levels of *FUCA1* (Angus heifers) and *FUCA2* (all animals) in the ipsilateral infundibulum linked to an upregulation of *FUCA1* in the ipsilateral ampulla of LFC heifers, and suggests a positive regulation of fucosidase by ovulation; perhaps to guarantee fertilization and embryo development. On the other hand, quantification of FUCA1 protein did not differ between ipsilateral and contralateral segments. One possibility is that the total amount of FUCA measured was from the entire oviduct, and not only from the secreted protein present in the OF.

After identification of fucose as a sperm binding site [29], Ignotz and collaborators [28] identified the location of the receptors, demonstrating the participation of four annexins in bovine sperm binding, ANXA1, ANXA2, ANXA4, and ANXA5, which are present in the cilia of the oviductal isthmus epithelium. The present study also showed the presence of all annexins in the ampulla [28], which indicates the continuous sperm binding to ampulla epithelial cells [70–72]. Moreover, ANXA4 shows ion and water regulation movement across human endometrium cells [73]. Higher levels of *ANXA2* and *ANXA4* in the ipsilateral infundibulum and ampulla, respectively, could be involved in sperm binding and/or controlling ion and water movement regulation across oviductal cells. The upregulation of ion and water movement in the ipsilateral oviduct by high levels of ANXA4 could explain the results of Kolle *et al*. [16]; they observed a thicker wall of the ipsilateral oviduct, which is more edematous and transparent than the contralateral oviduct (Fig 10).

Another important function of the oviduct is the transportation of gametes to the fertilization site and embryos to the uterus [74]. This is possible due to the presence of cilia beats and the contraction of the oviductal smooth muscle [75, 76]. Local production of prostaglandins (PTG), endothelin-1 (END-1), ANGII, and vascular endothelial growth factor (VEGF) is involved in the control of oviduct transport [21, 34, 35, 77, 78]. The association of prostaglandin F2α (PGF2α) and prostaglandin E_2_ (PGE_2_) results in a rhythmic control of contracting and relaxing smooth muscle cells, respectively [79]. Higher levels of PTGs and END-1 are present in the ipsilateral oviduct side from the developing dominant follicle and in the ovulation side [80], which increases amplitude and frequency of contractile activity in the oviduct [20, 37]. PGE_2_ receptors are involved in ciliary beating control, and the presence of PTGER2 and PTGER4 on epithelial cell surface was shown to support the stimulatory effect of PGE_2_ on CBF in hamster oviducts [81] and human uterine tube [82]. Additionally, functional studies showed that ANGII had a stimulatory action on CBF in human uterine tube [25]. In the present study, ipsilateral infundibulum presented higher levels of *VEGF* and *PTGER4* in all animals, and *ACE* in LFC heifers, when compared to the contralateral side. These results corroborate the stimulatory effect of an LH and E_2_ combination (hormonal profile of peri-ovulatory stage) on mRNA expression of *VEGF* [35]. *VEGF* expression could upregulate the biosynthesis of PGTs, END-1, and ACE in the infundibulum to stimulate CBF, as it does not possess smooth muscle [83], and the CBF has a positive impact on ovum pick-up and transport to the fertilization site. Moreover, there was an upregulation in the ipsilateral ampulla of *PTGS2* and *END1* levels, suggesting a positive influence on transportation in the ampulla (Fig 10).

In contrast to the infundibulum and ampulla, gene expression in the isthmus showed an opposite behavior. *VEGF* levels in LFC heifers and *KDR* levels (VEGF receptor) in all animals were lower in the ipsilateral isthmus, when compared to the contralateral side. Wijayagunawardane *et al*. [35] described a negative feedback mechanism of VEGF on its own system. Combined with LH and E_2_, VEGF blocks the stimulatory effect of LH and E_2_, and downregulates the oviductal VEGF system after ovulation. This is to suppress oviductal contraction to safely and slowly transport the embryo to the uterus at the required time [35]. Regulation of the VEGF system in the isthmus is possibly due to preparing this segment for receiving the presumable zygote and guaranteeing the correct speed of transport to the uterus (Fig 10).

Therefore, the answer to the initial question of this study (if the gene expressions of Aberdeen Angus and Nelore heifer oviducts are modulated by the antral follicular count) is partially minimal in our experimental context. It appears that the key factor in modulating the genes reported as potentially responsible for oviductal transport, sperm reservoir formation, monospermy control, and gamete interaction, one day after ovulation, is the differential microenvironment between ipsilateral and contralateral sides. Paracrine and autocrine factors produced by the preovulatory follicle or ovulation products might be responsible for modifying the oviductal microenvironment in preparation of the environment for fertilization. These factors would control the gene expression in the infundibulum and ampulla to guarantee correct gamete and embryo transport, polyspermy control, and gamete interaction. Therefore, the differential fertility capacity of different cattle breeds and AFCs might present a minimal impact on oviductal gene expression and might not be the principal component involved in bovine oviductal function. But our results support further studies to maximize understanding of the impacts of the cattle breeds and the AFC in the bovine oviduct physiology and function.
